# Robotic left-stapled total intracorporeal bowel anastomosis versus stapled partial extracorporeal anastomosis: operative technical description and outcomes

**DOI:** 10.1007/s00464-022-09048-6

**Published:** 2022-01-21

**Authors:** Hannah M. Hollandsworth, Kevin Li, Beiqun Zhao, Benjamin Abbadessa, Nicole E. Lopez, Lisa Parry, Sonia Ramamoorthy, Samuel Eisenstein

**Affiliations:** Department of Surgery, Division of Colon and Rectal Surgery, John and Rebecca Moores Cancer Center, University of California San Diego, 3855 Health Sciences Dr. #0987, La Jolla, CA 92037 USA

**Keywords:** Intracorporeal, Colon anastomosis, Robotic surgery, Colorectal cancer

## Abstract

**Background:**

Although there is extensive literature on robotic total intracorporeal anastomosis (TICA) for right colon resection, left total ICA using the da Vinci Xi robotic platform has only been described in short case series previously. In this study, we report on the largest cohort of robotic left total ICA, provide a description of our institution’s techniques, and compare outcomes to robotic left partial extracorporeal anastomosis (PECA).

**Methods:**

Patients who underwent robotic left colectomy for any underlying pathology from July 1, 2016 through April 30, 2020 were identified by procedure code. A technical description is provided for two unique techniques performed at our institution. Outcomes included operative time, length of stay, supply cost, post-operative ileus, post-operative morbidity and mortality and need for complete mobilization of the splenic flexure.

**Results:**

From a review of our institution’s data, 83 robotic TICA cases were identified and 76 robotic PECA cases were identified. Common procedures included low anterior resection, sigmoidectomy, left hemicolectomy, and rectopexy with resection. TICA was associated with significantly shorter intraoperative time compared to PECA.

**Conclusions:**

Our series shows that TICA is a safe and feasible technique that does not increase the risk of adverse outcomes. Using either the anvil-forward or anvil-backward technique, we were able to reliably reproduce this method in a total of 83 patients undergoing left colon resection for either benign or malignant diseases.

**Supplementary Information:**

The online version contains supplementary material available at 10.1007/s00464-022-09048-6.

With the transition of colorectal surgery to more minimally invasive techniques, including widespread use of robotic platforms, new techniques for creation of anastomoses have been evolving. When the transition to laparoscopic procedures from open procedures occurred, studies demonstrated superior outcomes after intracorporeal anastomosis (ICA) compared to extracorporeal anastomosis (ECA), including improved post-operative pain and decreased length of stay (LOS) [1]. Additional studies have demonstrated improved outcomes with laparoscopic ICA compared with laparoscopic-assisted colectomy with ECA [2, 3].

Prior case series have identified robotic ICA as a feasible technique after colonic resection, but the majority of the literature described right-sided ICA techniques. In one study, looking at right-sided ICA for malignancy, the authors found that the intracorporeal technique is a technically feasible and safe option for bowel anastomosis after right colectomy [4]. Another case study identified a robotic intracorporeal technique for anastomosis after resection of a splenic flexure tumor [5].

To date, most left-sided anastomoses are performed at least partially extracorporeally, placing the anvil into the bowel through an extraction site. This is potentially limiting as it can place tension on the mesentery of the bowel which has been hypothesized to increase the rate of postoperative ileus [1, 6] and may also potentially increase the need for the surgeon to further mobilize the bowel to exteriorize. It also potentially alters the incision pattern that the surgeon would use for the procedure as the extraction site needs to be reached easily by the left colon. Often times the robot is undocked once the bowel is exteriorized and the anastomosis is completed either laparoscopically or by redocking the robot, both of which could potentially increase the length of the procedure.

Further pilot studies have discussed robotic techniques for left-sided colorectal disease, including a case series describing robotic sigmoidectomy for diverticulitis that included robotic total ICA using the da Vinci Xi robotic platform [6]. This study demonstrated feasible technique with robotic total ICA with short post-operative LOS and minimal post-operative complications [6]. Although this study introduced the concept of the robotic left total ICA, it is limited by the small number of cases.

At our institution, we have 5 colorectal surgeons that perform da Vinci Xi robotic left colectomies. Three out of the 5 colorectal surgeons utilize a variation of robotic left total ICA, which allows us to evaluate a larger number of cases. In this study, we attempt to identify the largest case series of robotic left total ICA to date and compare outcomes with robotic left partial ECA. We also provide technical descriptions of two unique operative techniques for left total ICA.

## Materials and methods

### Operative selection

Patients were recruited from our institution between July 2016 and June 2020 with any colorectal diagnosis requiring low anterior resection, left hemicolectomy, or colostomy takedown that would require a low left-sided anastomosis. We included any disease process, including inflammatory bowel disease, colon cancer, diverticulitis, rectal cancer, or colonic stricture. Pre-operatively, all patients received a combination of mechanical and antibiotic bowel preparation.

### Patient positioning

The patient is placed in a supine position on the operating room table with both arms tucked and legs in modified lithotomy position using Yellowfin Stirrups (Allen Medical, Acton, MA). The entire abdomen is prepped with standard sterile surgical draping.

### Access and port placement: proctectomy

If the patient is to have an ostomy, we start by making the ileostomy site, which was marked by the ostomy nurse preoperatively. Through this site, we place a wound protector with a laparoscopic cap (Applied Medical, Rancho Santa Margarita, CA). If the patient is not to have an ostomy, we gain entry with a Veress needle or an optiview trocar just below Palmer’s point on the left side of the abdomen.

An 8 mm robotic port is placed through the laparoscopic cap, which will be used for the camera. The abdomen is insufflated using an AirSeal insufflation management system (ConMed, Utica, NY). Two 8 mm robotic ports are placed on the left side. A 12 mm robotic port is placed in the lateral RLQ. A 5 mm AirSeal port is placed in the RUQ for use as an assist port (ConMed, Utica, NY).

If there is no plan for a stoma, an 8 mm port will be placed in the right mid abdomen and the rightmost trocar will be used for the extraction site. A transverse, 3 cm muscle-splitting incision is made at this point in the procedure and capped wound protector is placed through with the 12 mm port in situ. If the surgeon is equivocal at the beginning of the procedure as to whether a stoma will be made at the end, often times ports will be placed without capped wound protector and the extraction site/ostomy site will be made later in the procedure when the surgeon has made their decision.

**Fig. 1:**
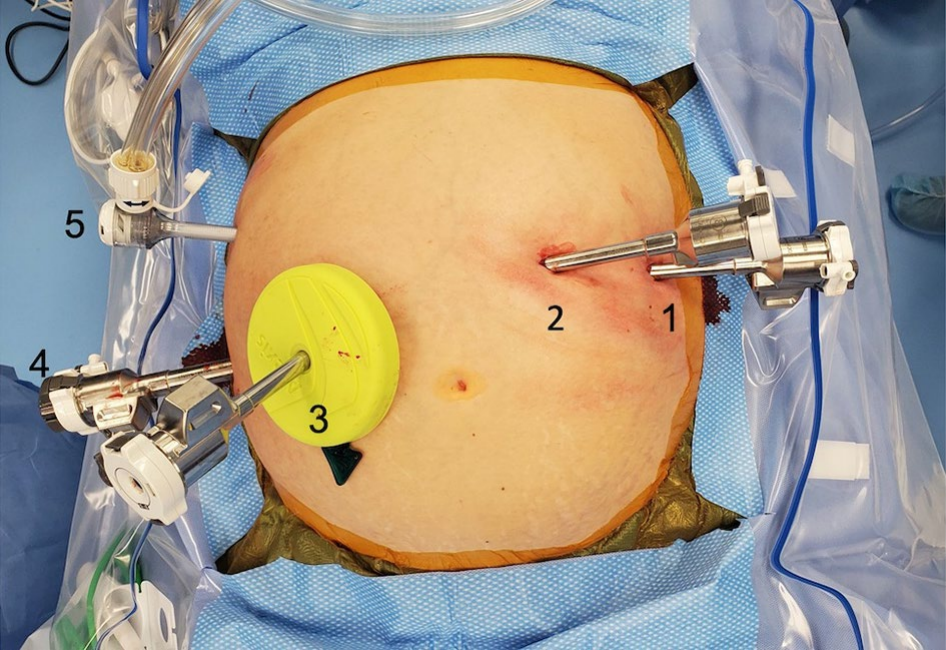
Trocar placement and instruments used for proctectomy. (1) 8 mm port, small grasping retractor. (2) 8 mm port, tip-up double-fenestrated grasper. (3) 8 mm port, robotic camera and future ileostomy site. (4) 12 mm port, hook with cautery, vessel sealer. (5) 5 mm laparoscopic assist port

The patient is placed in steep Trendelenberg. The da Vinci Xi robot is docked on the patients left side with the instruments on the boom facing toward the patient’s pelvis. A hook with cautery is initially used in the RLQ 12 mm port, a tip-up double-fenestrated grasper is introduced to the left medial 8 mm trocar, and a small grasping retractor is introduced into the left lateral 8 mm trocar (Fig. 1). Throughout the case, the hook cautery in the 12 mm port is exchanged for the vessel sealer, clip applier, and robotic stapler as needed.

### Access and port placement: sigmoidectomy

If the patient is to have an ostomy, we start by making the ileostomy site, which was marked by the ostomy nurse preoperatively. Through this site, we place a wound protector with a laparoscopic cap (Applied Medical, Rancho Santa Margarita, CA). If the patient is not to have an ostomy, we gain entry with a Veress needle at Palmer’s point on the left side of the abdomen and then use an optiview to enter in the right upper quadrant.

An 8 mm robotic port is placed through the laparoscopic cap, which will be used for the camera. The abdomen is insufflated using an AirSeal insufflation management system (ConMed, Utica, NY). Trocars are placed vertically with a slight angle so that the lower trocar is lateral to the uppermost one to help facilitate mobilization of the splenic flexure if necessary. 2 8 mm trocars are placed in the right upper abdomen, and a 12 mm trocar is placed in the RLQ.

If there is no plan for a stoma, an 8 mm port will be placed in the right lower mid abdomen and the lowest trocar will be used for the extraction site. A transverse, 3 cm muscle-splitting incision is made at this point in the procedure and capped wound protector is placed through with the 12 mm port in situ. If the surgeon is equivocal at the beginning of the procedure as to whether a stoma will be made at the end, often times ports will be placed without capped wound protector and the extraction site/ostomy site will be made later in the procedure when the surgeon has made their decision.

**Fig. 2:**
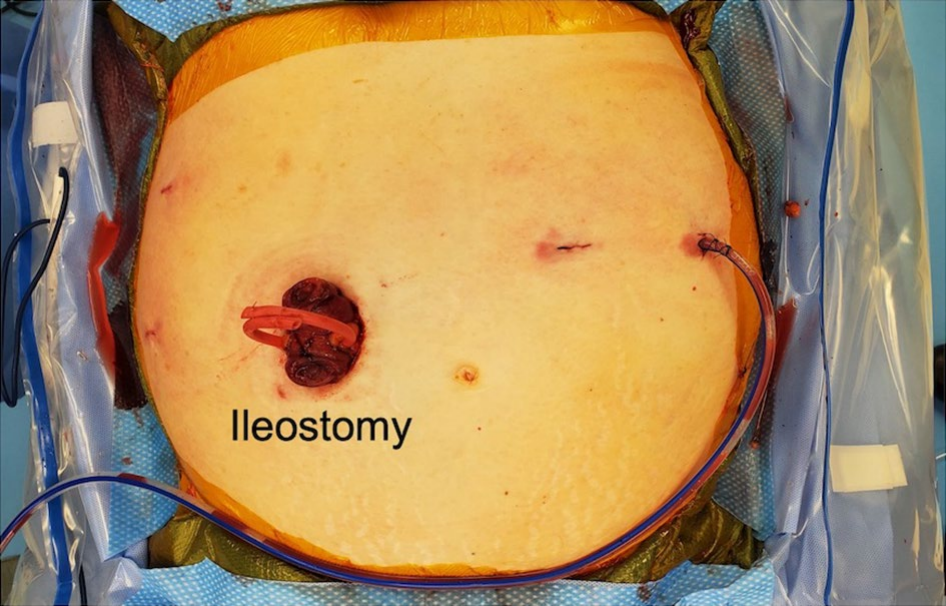
Conclusion of operation with loop ileostomy creation

The patient is placed in steep Trendelenberg. The da Vinci Xi robot is docked on the patients left side with the instruments on the boom facing toward the patient’s left. A hook with cautery is initially used in the RLQ 12 mm port, a tip-up double-fenestrated grasper is introduced to the trocar adjacent to the camera port, and and a small grasping retractor is introduced into the uppermost trocar (Fig. 2). Throughout the case, the hook cautery in the 12 mm port is exchanged for the vessel sealer, clip applier, and robotic stapler as needed.

### Dissection

The dissection is performed in standard fashion given the pathology. For proctectomies, if done for rectal cancer, we perform high ligation of the IMA. We also perform high ligation of the IMA for left-sided colon cancers. In general, no modification of the dissection is necessary for this technique, although it is important not to perform the proximal transection until after the distal transection is completed.

Once the distal transection is completed, the proximal transection point is identified and we take the mesentery using the vessel sealer to the wall of the bowel at this point. Perfusion is assessed by instilling indocyanine green using the Firefly technology on the robotic camera. Once we confirm our proximal transection point, it is marked with electrocautery. We then assess for length, ensuring this point will reach the anastomosis. If it does not, this is the time any lengthening procedures, such as mobilization of the splenic flexure should be performed. Once the anvil is in the bowel, ideally the anastomosis should be performed with minimal manipulation.

### ICA: anvil-forward technique

This technique will create a true end to end anastomosis. We begin by placing an EEA 29 anvil into the patient's abdomen through the lap cap which necessitates briefly undocking the robotic arm accessing through this port. We make an enterotomy distal to our transection point in the colon. Prior to placing the anvil into the bowel lumen, it is crucial to completely occlude the proximal bowel with the grasping retractor to prevent proximal migration of the anvil. We then place the anvil in the bowel head first, using the robotic clip applier without any clips as an anvil grasper, grasping the shaft. We then milk it proximal to the transection point. Once the anvil is proximal, we then transect the bowel at our marked point using the robotic stapler.

If the grasping retractor is well placed, the anvil will be apparent just anterior to the staple line and a small enterotomy is made anterior to the staple line to bring out the anvil. The specimen is then moved into the upper abdomen, and the anvil is brought in close proximity to the anastomosis. We then sequentially dilate the rectum with EEA sizers and bring the EEA 29 stapler up to a point in the rectum, just anterior to the staple line. The EEA stapler spike is brought out and married to the anvil using the empty robotic clip applier as an anvil grasper. This is inspected to ensure that there is no twisting of the bowel prior to firing the stapler. After firing the stapler, we remove the specimens and inspect the donuts to ensure that they are intact circumferentially. An air leak test is performed under saline in the pelvis using flexible sigmoidoscopy, and the anastomosis is inspected. If an ostomy is to be created, we use the ileostomy site as the extraction site for our specimen. If we are not creating an ostomy, the 12 mm port site is used as the specimen extraction site.

### ICA: anvil-backward technique

For this technique, a side-to-end stapled anastomosis with an EEA 29 stapler is performed. Again, we begin by placing an EEA 29 anvil into the patient's abdomen through the capped wound protector. We make an enterotomy proximal to our transection point in the colon. Next, the anvil is placed into the lumen, shaft side first, using the empty clip applier. The shaft is then immediately brought out through the side of the colon wall proximal to the transection point using electrocautery, taking care to leave enough room for the head of the anvil between the enterotomy and the transection point. During transection of the bowel, control of the anvil is maintained by grasping the shaft with the empty clip applier. The bowel is then transected just proximal to the colotomy using the robotic stapler, so the colotomy is included in the specimen. Anastomosis is performed similarly to the anvil-forward technique using the empty clip applier as an anvil grasper.

### Partially ECA

When we perform partially ECA, the operation starts with similar port placement and initial dissection. For adequate colonic length, the splenic flexure is often mobilized during this technique. Once mobilization of the colon is complete, the distal transection point is identified and the colon or rectum is divided with a robotic stapler at the chosen location. A 6 cm pfannenstiel incision is made, an Alexis wound retractor is placed and the colon exteriorized. The colon is then transected at a viable portion proximal to the IMA. A purse-string suture of 2–0 proline is placed and the anvil from a 29 EEA stapler introduced. The end of the colon is returned to the abdomen and the two ends of the stapler were mated, ensuring proper orientation, and the stapler fired. We then perform a leak test with the aid of flexible sigmoidoscopy.

### Patient selection

Under University of California San Diego (UCSD) IRB 191,476, we included da Vinci Xi robotic colorectal surgeries for any pathology that included left colon resection with colorectal or high coloanal anastomosis. Hand-sewn anastomoses and operations using the da Vinci Si system were excluded for consistency in the described technique.

### Outcomes

Outcomes included operative time (OT), LOS, and supply cost. 30-day mortality and post-operative morbidities, including postoperative ileus, anastomotic leak, surgical site infection (SSI), urinary tract infection (UTI), return to OR, return to ED, and readmission within 30 days post-operatively were assessed. Demographic information and comorbidities were also collected, including age, gender, ethnicity, smoking status, history of diabetes, and history of pelvic radiation.

### Statistical analysis

Statistical analysis was performed on SPSS version 24 (IBM Corporation, Armonk, NY). For continuous variables, normal distribution of the data was confirmed by the Shapiro–Wilk test. Outcomes for variables with normal distribution were reported as mean with standard deviation. Outcomes for variables without normal distribution are reported as median with 25th and 75th percentiles. For categorical variables, frequencies and proportions were reported. Continuous variable means with normal distribution for ICA and ECA were compared using student’s *t* test. Continuous variable medians with a non-normal distribution were compared using Mann–Whitney *U* analysis. Categorical variable outcomes for ICA and ECA were compared using chi-squared test. Significance was determined using *p* value 0.05 with two-tailed analysis.

## Results

We identified 83 da Vinci Xi cases where a left total ICA was performed and 76 cases where a left partially ECA was performed. Patient characteristics were largely similar among the two groups. Diagnoses include colon cancer, rectal cancer, diverticular disease, colonic stricture, rectal prolapse, and colonic fistula. Patients in the two operative groups were similar in age and BMI, and they had a similar proportion of female patients. They did not differ significantly in terms of ASA class or diagnosis. Similarly, they had similar rates of prior exposure to pelvic radiation therapy (Table 1).

**Table 1:** Demographic information and characteristics

	Total intracorporeal anastomosis (*n* = 83)	Partially extracorporeal anastomosis (*n* = 76)	*p* value^a^
Age (years), mean (SD)	56.53 (12.76)	58.99 (13.77)	0.245
Female sex, frequency (%)	43 (51.8)	43 (56.6)	0.633
BMI, mean (SD)	28.16 (5.43)	28.00 (6.71)	0.866
Race, frequency (%)			0.433
White	46 (55.4)	52 (68.4)	
Hispanic	22 (26.5)	13 (17.1)	
Asian	11 (13.3)	7 (9.2)	
Black	2 (2.4)	1 (1.3)	
Other	2 (2.4)	3 (3.9)	
ASA, frequency (%)			0.498
2	39 (47.0)	32 (42.1)	
3	43 (51.8)	44 (57.9)	
4	1 (1.2)		
Diagnosis, frequency (%)			0.835
Colon cancer	21 (25.3)	15 (19.7)	
Rectal cancer	29 (24.9)	27 (35.5)	
Diverticular disease	24 (28.9)	24 (31.6)	
Colonic stricture	5 (6.0)	3 (3.9)	
Rectal prolapse	1 (1.2)	2 (2.6)	
Colonic fistula	3 (3.6)	5 (6.6)	
History of pelvic radiation, frequency (%)	24 (28.9)	19 (25)	0.597

^a^Significance cut-off *p* < 0.05

TICA and PECA were utilized in low anterior resection, sigmoidectomy, left hemicolectomy, colostomy reversal, and rectopexy with colonic resection. The rates of operations involving either anastomotic technique were similar (Table 2). The PECA technique trended toward higher rates of splenic flexure mobilization, though that comparison did not result in statistical significance (TICA vs. PECA, 41% vs. 55%, *p* = 0.071). TICA was associated with a higher use of ICG compared to PECA (86% vs. 59%, *p* = 0.000). This did not, however, translate to improved anastomotic leak frequency (2.4% vs. 5.3%, *p* = 0.35). TICA was associated with shorter intraoperative time compared to PECA (197 min vs. 227 min, *p* = 0.002%). The two groups had similar reoperation rates (4.8% vs. 5.3%, *p* = 0.898), readmission rates (10.8% vs. 6.6%, *p* = 0.34), return to ED rates (15% vs. 15%, 0.998), and intra-abdominal infection rates (2.4% vs. 6.6%, *p* = 0.20). Although TICA was associated with shorter intraoperative time, the two techniques had comparable supply costs ($4426 vs. $4533; *p* = 0.52).

**Table 2:** TICA vs PECA

	Total intracorporeal anastomosis (*n* = 83)	Partially extracorporeal anastomosis (*n* = 76)	*p* value^a^
Operation, frequency (%)			
LAR	43 (51.8)	41 (53.9)	
Sigmoidectomy	39 (47.0)	33 (43.4)	
Colostomy takedown	1 (1.2)	2 (2.6)	0.355
Diverting ileostomy, frequency (%)	28 (33.7)	28 (36.8)	0.741
Splenic flexure mobilization, frequency (%)	34 (41.0)	42 (55.3)	0.071
Anastomosis height (cm), median (25th, 75th percentiles)	5.00 (4.00, 15.00)^b^	6.50 (4.00, 12.75)^c^	0.978
Use of ICG, frequency (%)	71 (85.5)	45 (59.2)	0.000*
Operative Time (min), mean (SD)	197.82 (55.15)	227.26 (63.50)	0.002*
Length of Stay (days), mean (SD)	4.25 (2.50)	4.67 (2.40)	0.948
ICU admission, frequency (%)	2 (2.4)	1 (1.3)	0.613
Post-operative Ileus, frequency (%)	8 (9.6)	9 (11.8)	0.798
Anastomotic leak, frequency (%)	2 (2.4)	4 (5.3)	0.346
Return to OR, frequency (%)	4 (4.8)	4 (5.3)	0.898
Return to ED within 30 days, frequency (%)	12 (14.5)	11 (14.5)	0.998
Readmission within 30 days, frequency (%)	9 (10.8)	5 (6.6)	0.343
Intra-abdominal infection, frequency (%)	2 (2.4)	5 (6.6)	0.201
Surgical site infection (SSI), frequency (%)	0 (0.0)	1 (1.3)	0.294
30-day mortality, frequency (%)	0 (0.0)	0 (0.0)	–
Supply cost ($), mean (SD)	4426.47 (965.11)	4533.84 (987.78)	0.517

^a^Significance cut-off *p* < 0.05

^b^Missing data; *n* = 43

^c^Missing data; *n* = 40

Subgroup analysis was done among patients undergoing TICA to compare the outcomes following the anvil-forward versus the anvil-backward technique (Table 3). There were 42 patients who underwent the anvil-forward technique, and 41 patients underwent the anvil-backward technique. There was a frequency of patients undergoing LAR, sigmoidectomy, and colostomy takedown. In either technique, a similar proportion of patients also received a diverting ileostomy. The anvil-backward technique trended toward a higher rate of splenic flexure mobilization (31% vs. 51%, *p* = 0.06). However, the anvil-forward technique was associated with a higher anastomosis height, though the absolute difference is relatively small (6.0 cm vs. 4.5 cm, *p* = 0.045%). The two techniques had similar operative times (191 min vs. 204 min, *p* = 0.29) and comparable rates of ICG use (81% vs. 90%, *p* = 0.23). ICG use varied due to date of the operation. In more recent operations, ICG has become a standard portion of the operation to assess perfusion of the proximal and distal bowel to be involved in the anastomosis. In rare incidences, further bowel resection was performed if perfusion was deemed inadequate. This was not found to be significant when compared to rate of anastomotic leak in this series. Among post-operative complications, the anvil-forward technique was associated with a higher rate of reoperation, (9.5% vs 0%, *p* = 0.04). The two groups had similar rates of return to ED within 30 days (12% vs. 17%, *p* = 0.50), 30-day readmission (12% vs. 10%, *p* = 0.75), and post-operative intra-abdominal infection rates (2.4% vs. 2.4%, *p* = 0.99). Each technique had a similar operative supply cost ($4,589 vs. $4,264; *p* = 0.15).

**Table 3:** Total intracorporeal anastomosis: Anvil-forward versus Anvil-backward technique

	Anvil forward (*n* = 42)	Anvil backward (*n* = 41)	*p* value^a^
Operation, frequency (%)			0.399
LAR	20 (47.6)	23 (56.1)	
Sigmoidectomy	22 (52.4)	17 (41.5)	
Colostomy takedown	0 (0.0)	2 (4.9)	
Diverting ileostomy, frequency (%)	17 (40.5)	11 (26.8)	0.189
Splenic flexure mobilization, frequency (%)	13 (31.0)	21 (51.2)	0.060
Anastomosis height (cm), median (25th,75th percentile)	6.00 (5.00, 15.00)	4.50 (3.00, 11.50)	0.045*
Use of ICG, frequency (%)	34 (81.0)	37 (90.2)	0.229
Operative time (minutes), mean (SD)	191.50 (56.72)	204.39 (53.40)	0.286
Length of stay (days), median (25th,75th percentile)	4 (3, 5.25)	4 (3, 5)	0.098
ICU admission (days), mean (SD)	1 (2.4)	1 (2.4)	0.986
Post-operative Ileus, frequency (%)	5 (11.9)	3 (7.3)	0.479
Anastomotic leak, frequency (%)	2 (4.8)	0 (0.0)	0.157
Return to OR, frequency (%)	4 (9.5)	0 (0.0)	0.043*
Return to ED within 30 days, frequency (%)	5 (11.9)	7 (17.1)	0.503
Readmission within 30 days, frequency (%)	5 (11.9)	4 (9.8)	0.753
Intra-abdominal Infection, frequency (%)	1 (2.4)	1 (2.4)	0.986
Surgical site infection (SSI), frequency (%)	0 (0.0)	0 (0.0)	–
30-day mortality, frequency (%)	0 (0.0)	0 (0.0)	–
Supply cost ($), mean, SD	4588.92 (1035.22)	4264.01 (873.40)	0.149

*Highlights the values which are considered significant based on *p* < 0.05

^a^Significance cut-off *p* < 0.05

## Discussion

Minimally invasive colon resections are associated with shorter LOS and post-operative pain compared to open resections. This association carries even to the method of anastomosis, where for right-sided resections ICA has superior outcomes compared to ECA, in terms of post-operative recovery and LOS. In this paper, we explored whether this association holds true for left-sided colonic resections. To our knowledge, this study represents the largest series evaluating total intracorporeal anastomosis (TICA) versus partial extracorporeal anastomosis (PECA) following left colon resections for both benign and malignant diseases.

Our series shows that TICA is a safe and feasible technique that does not increase the risk of adverse outcomes. Using either the anvil-forward or anvil-backward technique, we were able to reliably reproduce this method in a total of 83 patients undergoing left colon resection for either benign or malignant diseases. The choice to perform anvil-forward versus anvil-backward technique was surgeon dependent, and there were no differences in outcomes based on chosen technique. TICA is also transferable to multiple surgeons, as our group involved 5 surgeons performing this procedure. Indeed, robotic surgical procedures in general have a reproducible learning curve. However, TICA may not require a significant learning curve to attain proficiency among experienced colorectal surgeons with proficiency in robotic surgery. As a result, the technique may be adopted among different institutions. Further studies will be needed to assess a volume–outcome relationship for TICA in left colon resections.

Our data show that TICA is superior to PECA regarding intraoperative time. TICA saves time in that it requires less mobilization of the splenic flexure, and it does not require undocking the robot to perform the anastomosis. The time saving in our study was practically significant, with an average of 30 min of reduced operative time in TICA. Our study captured supply costs but did not factor the time cost of using the operating room. Further cost studies incorporating the operating room time cost would be required to assess whether TICA provides an absolute cost saving compared to PECA within the operating room.

Our subgroup analysis comparing the anvil-forward versus the anvil-backward techniques showed that either technique is safe and feasible. The anvil-backward technique may be favored in anastomosis occurring low in the pelvis, as it had on average a lower anastomosis height compared to the anvil-forward method. The anvil-forward method did have a higher rate of reoperation, though our series is limited by relatively small sample size, which calls into question whether the anvil-forward method is truly associated with reoperation. Therefore, among practitioners of TICA in left colon resections, either the anvil-forward or the anvil-backward technique is equally valid, and the choice may depend largely on surgeon’s preference and familiarity with the procedure.

Our study carries several limitations. Although it is the largest case series of TICA for left colon resections, it remains a relatively small series involving a total of 159 patients. As a result, it is underpowered to detect significant differences in post-operative outcomes. Although we were able to detect a difference in operative duration, we are unable to conclude that TICA is superior to PECA in terms of LOS or complication rates. In addition, this is a single institution case series, so while our results show that TICA is safe and feasible the same results may not be replicated in a separate institution. Our hope is that the technique may be readily reproduced in different institutions, as in our experience a relatively few numbers of cases was performed by each surgeon. Since this study acts as a technical description regarding the feasibility of technique for TICA, it did not explore complications associated with our specimen extraction site. Further prospective studies with a larger cohort should be performed to investigate complications associated with this technique. In this series, the use of ICG was varied, with ICG use being more common in more recent procedures. This limits the ability to adequately assess the use of ICG in avoiding the complication of anastomotic leak. In current practice, the use of ICG has become standardized in these operations at our institution.

Previous studies have questioned the economic value of the use of robotic technology in colorectal surgery, which is an important consideration when choosing the correct surgical technique [7]. In this study, they compared cost-effectiveness of robotic versus laparoscopic right hemicolectomy with ICA [7]. In our study, we aim to describe a technique for ICA for left-sided colectomy. For low anterior resections, the robot affords improved visualization and ability to perform pelvic dissections, whereas right colectomies are technically more feasible with a laparoscopic approach. Further studies should be performed to assess the cost-effectiveness of robotic left hemicolectomy with TICA compared to a laparoscopic approach.

TICA is a safe and feasible procedure that was reproduced among our group of colorectal surgeons. This finding has been validated in a recent series described from the Mayo Clinic, which also found that TICA is feasible for left-sided colectomies [8]. While we have been able to show that TICA offers shorter intraoperative time, further studies are needed to evaluate post-operative outcomes and to further assess the reproducibility among different institutions.

## Supplementary Information

Below is the link to the electronic supplementary material.

Supplementary file1 (MP4 475890 kb)
